# Simplifying functional network representation and interpretation through causality clustering

**DOI:** 10.1038/s41598-021-94797-y

**Published:** 2021-07-28

**Authors:** Massimiliano Zanin

**Affiliations:** Instituto de Física Interdisciplinar y Sistemas Complejos (IFISC) (CSIC-UIB), Campus UIB, 07122 Palma de Mallorca, Spain

**Keywords:** Complex networks, Cognitive neuroscience, Computer science

## Abstract

Functional networks, i.e. networks representing the interactions between the elements of a complex system and reconstructed from the observed elements’ dynamics, are becoming a fundamental tool to unravel the structures created by the movement of information in systems like the human brain. They also present drawbacks, one of the most important being the inherent difficulty in representing and interpreting the resulting structures for large number of nodes and links. I here propose a causality clustering approach, based on grouping nodes into clusters according to their similarity in the overall information dynamics, the latter one being measured by a causality metric. The whole system can then arbitrarily be simplified, with nodes being grouped in e.g. sources, brokers and sinks of information. The advantages and limitations of the proposed approach are discussed using a set of synthetic and real-world data sets, the latter ones representing two neuroscience and technological problems.

## Introduction

Born within complex networks theory^1,2^, the concept of *functional networks* has supposed a revolution in the way the study of complex systems has been approached in the last decade^3^. Firstly introduced in neuroscience^4^, functional networks are based on the hypothesis that relationships between the elements composing a system affect their respective dynamics, such that the dynamics is a *function* of the structure; the latter, and specifically, time series representing such dynamics, can then be used to infer the underlying connectivity. The connectivity structure of a complex system thus stops being information required to correctly understand the behaviour of the system, and becomes a result of the analysis (and of the behaviour) itself. Functional networks thus became the instrument of choice not only for studying those systems whose underlying connectivity structure is unknown; but also for understanding how such connectivity, i.e. the internal flow of information, adapts to external conditions.

The prototypical application of functional networks is probably the analysis of brain dynamics^4–7^. Starting from neuroimaging data, for instance recorded through electroencephalography (EEG), magnetoencephalography (MEG) or functional magnetic resonance imaging (fMRI), the resulting networks represent how information is distributed across different brain regions. This analysis can both be performed for an unguided dynamics (what is known as *resting state*) or during specific cognitive tasks; and can be used to compare healthy and pathological dynamics. Beyond neuroscience, functional networks have found applications in other fields like biology^8^, econophysics^9–11^, air transport^12–14^, or epidemiology^15^.

A problem inherent to functional networks is the complexity associated to their representation and interpretation. To illustrate, a typical functional network is composed of *N* nodes, and of weighted links between each pair of them (for a total of $L \propto N^2$ links). Even if some links are disregarded, e.g. using thresholds on the weights or statistical tests, sparse networks can still have link densities of the order of $5\%$; as a result, the graphical representation of a system of $N>100$ nodes and $L>500$ links is usually a seemingly unstructured cloud of points and lines. To make things even more complex, causality metrics yield links that are directional, with each node potentially being both at the sending and receiving ends of multiple links. Except for some very simple cases and very small networks, it becomes difficult to manually understand the role of each node. In other words, functional networks yield a very detailed representation of the trees; but at the same time they prevent visualising the global forest.

I here propose to overcome these interpretation challenges through the application of a novel causality clustering approach. The starting point is the hypothesis that functional networks we observe are the sum of two contributions: a main flow of information, and additional secondary flows. Note that these secondary flows may be inherent to the activity of the system, but may also be the result of observational noise and statistical false positives. A clearer representation could be obtained if these secondary causality links were deleted, or somehow excluded from the final representation. In order to achieve this, I firstly fix a global target causality pattern, i.e. a small graph where vertices represent clusters (or sets of nodes in the original network that share the same causality role) and edges main flows of information. Secondly, nodes of the network are assigned to the clusters in order to maximise the statistical significance of the pattern. To illustrate, in the simplest case of two clusters, only one asymmetric pattern is possible, with information flowing from the first to the second cluster. Nodes are then assigned to each cluster in order to maximise such causality relation; the result can easily be interpreted, as elements in the first cluster are mostly *forcing*, while those in the second are mostly *being forced*. This approach can easily be generalised to high-order patterns; and, while here I focus on the celebrated Granger causality^16^, it can in principle be used with any causality metric. In what follows, I propose a definition of such causality clusters, and demonstrate their applicability with a set of synthetic and real-world data sets, the latter ones representing two neuroscience and technological problems. I finally discuss the limitations of the method, especially regarding its computational cost, and propose approximated solutions.

## Results

### The Granger causality test

The Granger causality test^16^, developed by the economy Nobel Prize winner Clive Granger (possibly leveraging on related concepts proposed one decade earlier by Norbert Wiener^17^), is one of the best well-known metrics for assessing *predictive causality*^18^ between elements composing a system. For this reason, and without lack of generality, this test has been chosen to illustrate the method; still, any other equivalent causality measure can be used, as will be discussed in the conclusions.

The Granger test is based on the idea that knowing the past dynamics of the *causing* element must help predicting the future dynamics of the *caused* element, as by definition the latter is partly defined (or constrained) by the former. Since its introduction, this test has been applied to uncountable problems, from economics^19–22^, engineering^23^, sociology^24^, biology^25^ or neuroscience^26–28^. While a full discussion of the hypotheses and limitations of the test are beyond the scope of this work, for the sake of completeness, its basic mathematical formulation is reported below.

Suppose that the dynamics of two elements *A* and *B*, composing a larger system, is described by two time series $a_t$ and $b_t$. Further suppose that these time series fulfil some basic conditions, including being stationary and regularly sampled. Using the notation originally introduced by Granger^16^, *B* is said to “Granger-causes” *A* if:

1 $$\begin{aligned} \sigma ^2 ( A | U^- ) < \sigma ^2 ( A | U^- \backslash B^- ), \end{aligned}$$

where $\sigma ^2 ( A | U^- )$ stands for the error (i.e. the standard deviation of residuals) in forecasting the time series *A* using the past information of the entire universe *U*, i.e. of all elements composing the system; and $\sigma ^2 ( A | U^- \backslash B^- )$ the error when the information about time series *B* is discarded. When the forecast is performed through an autoregressive-moving-average (ARMA), two models are fitted on the data, respectively called the restricted and unrestricted regression models:

2 $$\begin{aligned} a_t=  C \cdot a_{t-1}^m + \varepsilon _t, \end{aligned}$$

3 $$\begin{aligned} a_t=  C' \cdot \left( a_{t-1}^m \oplus b_{t-1}^m \right) + \varepsilon '_t. \end{aligned}$$

*m* here refers to the model order, the symbol $\oplus$ denotes concatenation of column vectors, *C* and $C'$ contain the model coefficients, and $\varepsilon _t$ and $\varepsilon '_t$ are the residuals of the models. Equation (1) is then usually written as $\sigma ^2( \varepsilon '_t ) < \sigma ^2( \varepsilon _t )$. As a final step, an F-test is performed to assess the statistical significance of this inequality.

As a final note, the reader should note that, while the test is commonly called Granger causality, it does not necessarily measure true causality—as notably was highlighted by Clive Granger himself^29^. A more precise definition should be based on concepts like *predictive causality*^18^, as it assesses how one time series can be used to predict a second one; directed lagged interactions between joint processes; or the quantification of information transfer across multiple time scales. In spite of this, and for the sake of simplicity, the relationships detected by this test will here be called *causal*.

### Calculating causality clusters

Let us suppose a set of *N* elements, where the *i*-th element is described by a time series $x_i(t)$. No special requirements are associated to these time series, beyond the standard ones for the calculation of a Granger test, i.e. stationarity, equal and regular sampling, and absence of missing values. A standard functional network analysis, as for instance common in neuroscience^26,27^, entails reconstructing an *adjacency matrix* *A*, of size $N \times N$, where each element $a_{i, j}$ is equal to one if the Granger test between time series $x_i$ and $x_j$ yields a statistically significant result.

I here propose a different approach, based on finding the best clustering of these elements according to a pre-defined causality motif. Let us define *C* as the number of clusters to be considered, and ${\fancyscript {P}}$ a function that assigns each of the *N* elements to one of the *C* clusters. Each cluster is then described by a time series *y*(*t*), which is the sum of all time series of the elements belonging to that cluster. Additionally, *M* is a matrix of size $C \times C$ that defines the desired connectivity motif; its meaning is that of an adjacency matrix, such that the element $m_{i, j}$ is equal to one if a significant Granger causality is expected between the time series of clusters *i* and *j*.

Let us further denote with $pV_{i,j}$ the *p*-value yielded by the Granger test when applied to the time series corresponding to clusters *i* and *j*. As standard in statistics, this *p*-value is the probability of finding a causality effect in the null model at least as extreme as the one actually observed. As natural, $1 - pV_{i,j}$ is the probability of not finding a causal effect in the null model larger that what observed. This interpretation of the *p*-value can be extended to the case of three or more clusters. Specifically, suppose the case of three clusters *i*, *j* and *k*; and that a Granger causality is expected between *i* and *j*, but is not expected between *j* and *k*. The product $pV_{i,j} \cdot ( 1 - pV_{j,k} )$ would then be proportional to the probability of observing both a false causality between *i* and *j*, and a false lack of causality between *j* and *k*. Note that this probability of observing a false causality, also called False Positive Risk (FPR), and the *p*-value are not equivalent, as the former also depends on the prior probability of having a real effect^30^; for the sake of simplicity, we here consider that the latter probability is constant throughout all the tests, thus making FPRs and *p*-values proportional. The aforementioned product can easily be extended to all possible pairs of clusters, as:

4 $$\begin{aligned} J = \prod _{i=1} ^C \prod _{j \ne i, j=1} ^C \left[ 1 - pV_{i,j} + m_{i, j} ( 2 pV_{i,j} - 1 ) \right] , \end{aligned}$$

with $pV_{i,j}$ being the *p*-value yielded by the Granger test when applied to the time series corresponding to clusters *i* and *j*; and $m_{i,j} = 1$ if a Granger causality is expected between clusters *i* and *j*, and zero otherwise. *J* can thus be understood as the probability of finding the connectivity motif *M* under the assumption that the null hypothesis is correct, or *M*’s statistical significance. The goal of the clustering analysis is then to find the mapping ${\fancyscript {P}}$ that minimises the value of *J*.

A simple example can further help illustrating the meaning of *J* and of its optimisation. Let us fix $C = 2$ and $$M = \big ({\begin{matrix} 0 &{} 1\\ 0 &{} 0 \end{matrix}}\big )$$. For $i = 1$ and $j = 2$, *m* is equal to 1, and the factor in Eq. (4) simplifies to $pV_{1, 2}$; on the other hand, for $i = 2$ and $j = 1$, one has $m_{2, 1} = 0$ and the summand becomes $1 - pV_{2, 1}$. This implies that *J* is minimised by both small values of $pV_{1, 2}$ and large (i.e. close to one) values of $pV_{2, 1}$. Optimising *J* is thus equivalent to finding the assignation of elements to the two clusters such that the causality between clusters $1 \rightarrow 2$ is maximised, while the causality $2 \rightarrow 1$ is minimised. In other words, the original *N* elements are distributed among the two clusters such that, globally, elements in the first are forcing those in the second.

A more complex example involves setting $C = 3$ and $$M = \big ({\begin{matrix} 0 &{} 1 &{} 0\\ 0 &{} 0 &{} 1 \\ 0 &{} 0 &{} 0 \end{matrix}}\big )$$. In this case, minimising *J* is equivalent to distribute the original *N* elements among three clusters, such that elements in the first only cause elements in the second clusters, and these force elements in the third. Elements in the first cluster are thus *net causes*, while those in the third are *net caused*. Finally, elements in the second cluster can be considered as *broker* or intermediate nodes, passing information from the first group to the third.

Before applying this idea to synthetic and real data, it is important to stress a couple of aspects. First of all, the clustering here defined is based on global causalities, as opposed to micro-scale ones. For instance, in the case $$M = \big ({\begin{matrix} 0 &{} 1\\ 0 &{} 0 \end{matrix}}\big )$$, it may be possible to find two elements *i* and *j*, respectively assigned to clusters 2 and 1, with the former causing the latter—i.e. the opposite direction than the one defined by *M*. This is possible, provided *i* is caused by multiple elements in the first cluster, and *j* is also causing other elements in the second cluster. In other words, clusters 1 and 2 are respectively *net sources* and *net receivers* of causality relations, but not *absolute* ones.

Secondly, this clustering is not equivalent to one obtained by simply counting the number of inbound and outbound causality links. Specifically, an element being weakly forced by two elements and strongly forcing a fourth one may end belonging to the first cluster, as the outbound causality may contribute more than the two inbound ones. On the other hand, one can imagine an element forcing a large group of elements, but in a very weak way—i.e. with a *p*-value not passing the significance level. When these latter elements are merged into a single cluster, their time series are summed, and the result may become a statistically significant causality relation. In synthesis, the final clustering solution cannot be inferred by the causality calculated between pairs of elements.

Thirdly, and as a direct consequence of the previous point, the calculation of the optimal mapping ${\fancyscript {P}}$ is a highly computationally costly process, as all possible combinations have to be checked—yielding a complexity of $O(C^N)$. Still, approximate solutions can be found, as will be discussed below.

**Figure 1:**
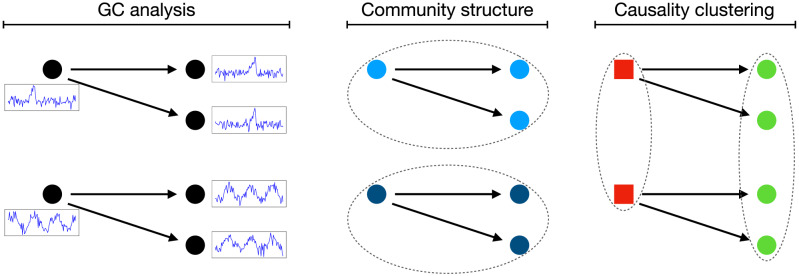
Causality clustering vs. network community structure. (Left) Toy system composed of six elements, with arrows representing the Granger causality relationships between them. (Center) Assignation of the elements to communities, following network theory’s definition. (Right) Assignation of the elements to two clusters, as proposed in this work.

Finally, obtaining ${\fancyscript {P}}$ is not equivalent, but instead complementary, to community detection in complex networks^31,32^. To illustrate this point, suppose a simple system composed of six elements, two of them forcing the remaining four—see Fig. 1 Left for a graphical depiction, with arrows representing statistically significant Granger tests. When the resulting structure is interpreted as a network, two communities (actually corresponding to two independent components) are identified, respectively comprising the top and bottom nodes—see the central panel. This follows the definition of communities as sets of nodes strongly connected between themselves. On the other hand, the approach here proposed would yield the structure depicted in the right panel, with the two left nodes (i.e. the *net sources* of the causality, in red) belonging to the first cluster, and the four right ones (i.e. the *net receivers*, in green) to the second. In other words, while community detection in complex networks focuses on identifying groups of nodes interacting strongly between them, the present approach focuses on identifying groups of nodes performing a similar role, independently on whether they belong to the same component or not.

### Validation on synthetic data

In order to test the validity of the proposed clustering concept, this is here firstly applied to a set of synthetic data; these present the advantage of being clearly defined, and of allowing controlling the strength of the causality relations between pairs of elements.

I here consider a system composed of *N* linearly coupled elements, such that their dynamics is defined as $x_i(t) = \xi$ for $i = 1, \ldots , N/2$, and $x_i(t) = \xi + \gamma x_{i-N/2}(t-2)$ for $i = N/2+1, \ldots , N$, with $\xi$ representing random numbers independently drawn from a normal distribution ${\mathcal {N}}(0, 1)$. In other words, the first *N*/2 elements have a completely random (and independent) dynamics; while the second half also have an independent dynamics, but are also linearly forced by the first group with a strength $\gamma$ and a time lag of 2. The advantage of this configuration is that the optimal solution is known, with the first and second half of the elements respectively belonging to the first and second clusters, while full control is retained over the strength of the causality relation.

**Figure 2:**
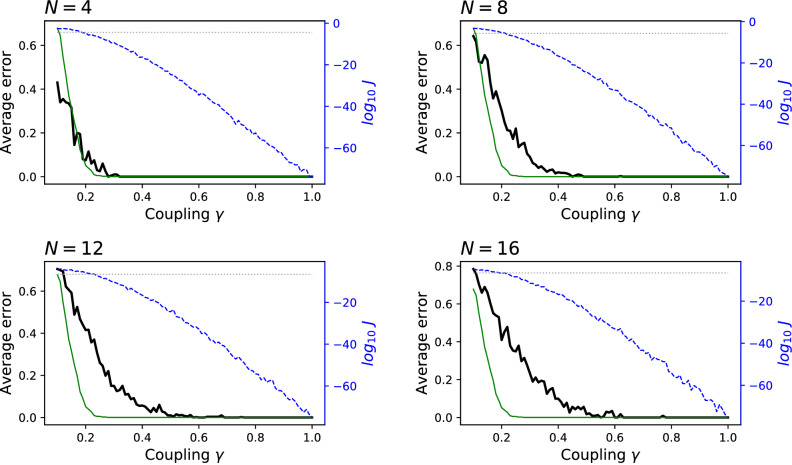
Significance of the clustering for synthetic data and $C=2$. Each panel reports, for different values of *N* and as a function of the coupling $\gamma$: the average error of the best clustering, compared with the real cluster assignation (black lines, left axes); fraction of times the Granger causality test fails to detect a statistically significant result (green lines, left axes); average of the $\log _{10} J$ of the solution (blue dashed lines, right axes); and average *J* obtained for uncorrelated time series (grey dotted horizontal lines, right axes). See main text for details on the reconstruction of the synthetic data.

The four panels of Fig. 2 present the results for $N=4$, 8, 12 and 16. Specifically, the black lines correspond to the average error (fraction of misassigned elements) as a function of the coupling $\gamma$; and the blue dashed lines the $\log _{10}$ of the average *J* (right axis). As a reference, the thin green lines further depict the fraction of Granger causality tests failing to detect a statistically significant result (with $\alpha = 0.01$) between $x_1$ and $x_{N/2+1}$, i.e. on a single pair of time series, also as a function of $\gamma$; and the dotted horizontal grey line the average *J* obtained for uncorrelated time series (right axis). It can be appreciated that the exact solution is always recovered; yet, this comes at the cost of a larger value of the coupling $\gamma$ when the system includes a large number of elements. The value of *J* can also be used as an estimator of the validity of the found solution: when the blue and grey lines intersect, i.e. when *J* gets below what expected in uncorrelated time series, the error drops to $\approx 0.2$.

The same analysis can also be performed for the case of three clusters, i.e. with $$M = \big ({\begin{matrix} 0 &{} 1 &{} 0\\ 0 &{} 0 &{} 1 \\ 0 &{} 0 &{} 0 \end{matrix}}\big )$$. In this case, the dynamics of the system is set as:

5 $$\begin{aligned} x_i(t) = {\left\{ \begin{array}{ll} \xi &{} i = 1, \ldots , N/3\\ \xi + \alpha x_{i-N/3}(t-2) &{} i = N/3+1, \ldots , 2N/3\\ \xi + \alpha x_{i-N/3}(t-2) &{} i = 2N/3+1, \ldots , N. \end{array}\right. } \end{aligned}$$

**Figure 3:**
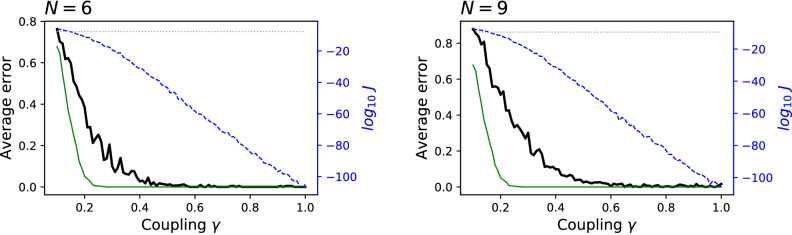
Significance of the clustering for synthetic data and $C=3$. Meaning of lines and axes is the same as in Fig. 2. See main text, and specifically Eq. (5), for details on the reconstruction of the synthetic data.

In other words, the first third of the elements only force the dynamics of the second third, and these, in turns, force the dynamics of the last third. The numerical results for $N=6$ and $N=9$ are depicted in Fig. 3. Note that, in this case, the maximum lag allowed in the calculation of the Granger causality has been fixed to 3, in order to avoid the detection of a relationship between the first and third groups—which are indirectly related by a time lag of 4. The same behaviour is observed, i.e. the exact solution is recovered, provided a large enough coupling is present.

**Figure 4:**
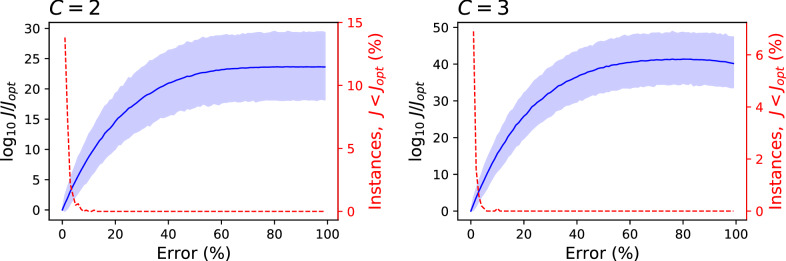
Relationship between *J* and the quality of the clustering. Both panels report the average (solid blue lines) and the $10{-}90$ percentile interval (blue bands) of the metric $\log _{10} J / J_{opt}$; see main text for definition. The red dashed lines (right Y axes) report the percentage of random realisations for which $J < J_{opt}$. Left and right panels respectively correspond to $C=2$ and $C=3$.

The relationship between *J* and the quality of the solution can easily be tested using these two synthetic models. Specifically, Fig. 4 considers a system composed of 100 elements, causally connected between them ($\gamma = 0.5$) and organised in two (left panel) and three (right panel) clusters. Given that the exact solution is known by construction, it is possible to calculate the corresponding $J_{opt}$. Subsequently, the cluster assignation of a random subset of nodes can be changed, such that they are assigned to a random cluster different from the initial one, therefore obtaining a worse solution and a larger *J*. Figure 4 finally reports the difference between the latter and the former, i.e. $\log _{10} J / J_{opt}$. Specifically, the solid blue lines correspond to the average of the metric over $10^4$ random realisations, and the transparent bands to the $10{-}90$ percentiles. It can be appreciated that *J* increases as the solution get worse, and that only a small percentage of wrong solutions have a *J* less than $J_{opt}$—see red dashed lines (right Y axes) in both panels.

**Figure 5:**
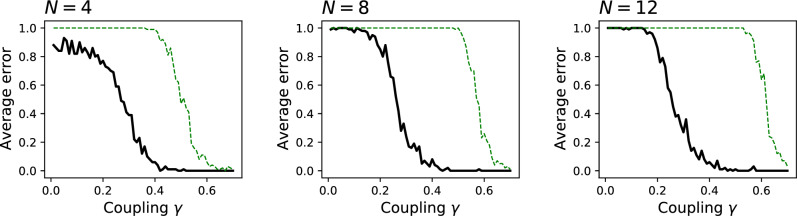
Improved sensitivity due to merging. The three panels report, for $N = 4$, 8 and 12, and as a function of the coupling $\gamma$: the average error of the best clustering, compared with the real cluster assignation (black solid lines); and the fraction of times the Granger causality test fails to detect a statistically significant result between all elements of the first cluster and those of the second (green dashed lines).

Beyond being able to yield a simpler representation of the causal interactions, the proposed method presents the advantage of detecting weak causalities, provided they interact in a constructive way when elements are merged in clusters. Once again, let us consider the case of a set of linearly coupled elements, whose dynamics is given by $x_i(t) = \xi$ for $i = 1, \ldots , N/2$, and $x_i(t) = \xi + \gamma x_{i-N/2}(t-2)$ for $i = N/2+1, \ldots , N$. If $\gamma$ is small enough, i.e. if the time series are very noisy, a Granger causality test may fail in detecting the coupling between pairs of elements. On the other hand, when elements are clustered together, the noisy component can cancel out, yielding a clearer picture of the interactions. To test this, Fig. 5 reports the results for $N=4$, 8 and 12. Specifically, the black solid lines correspond to the error (in terms of fraction of misclassified elements) of the proposed method. Green dashed lines, on the other hand, represent the fraction of times a simple pairwise Granger causality is not able to detect all correct relationships, i.e. $x_1 \rightarrow x_{N/2+1}$, $x_2 \rightarrow x_{N/2+2}$, $\ldots$, $x_{N/2} \rightarrow x_{N}$. As hypothesised, coupling must be fairly strong to get an exact Granger causality picture, while the proposed method is able to recover the underlying structure starting with $\gamma = 0.2$.

### Application: EEG functional networks

**Figure 6:**
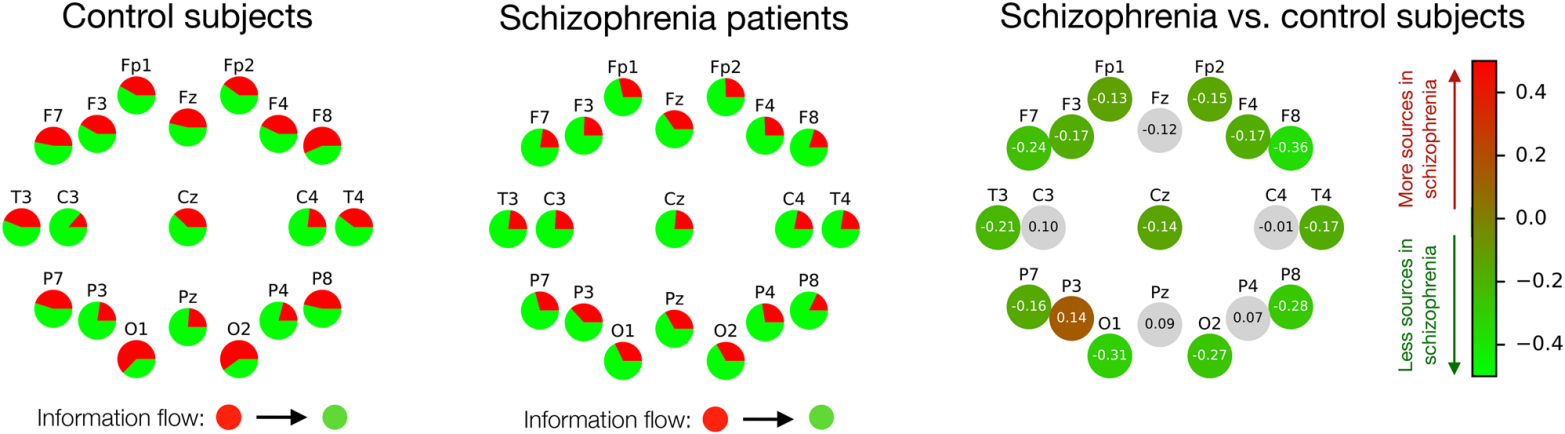
Causality clustering of EEG brain signals. The first two panels report the role of nodes (EEG sensors) for control subjects (left) and Schizophrenia patients (center). The red (green) part of each node represents the fraction of times that node has been classified in cluster 1 (respectively, 2), hence being a source (respectively, a sink) of information. The right panel represents the difference between Schizophrenia patients and control subjects, with green shades indicating nodes that are less frequently sources of information in patients. Grey nodes indicate no statistically significant differences between both groups at $\alpha = 0.01$.

As a first example of a real-world application, I here consider a set of time series representing the electric activity of the brain (recorded through electroencephalography, EEG) for a set of patients suffering from Schizophrenia and matched control subjects—for details on the trials, time series and processing, see “Materials and methods”. The results for $C=2$ are represented in Fig. 6, including control subjects (left) and Schizophrenia patients (center). Specifically, each circle represents an EEG sensor, with the corresponding name reported on top of it. Additionally, each circle is a pie graph, in which the red and green parts respectively represent the fraction of trials in which that sensor was classified in the first or second cluster. In other words, the larger the red part, the more frequently that sensor has been classified as a source of information—or as a *forcing* node, in the Granger causality sense.

Several interesting conclusions can be drawn. First of all, there is a marked symmetry between the left and right hemispheres, as is generally expected in a resting state^33^; at the same time, factors that are known to contribute to lateralisation, as e.g. handedness and sex^34,35^, were not reported in this data set and could therefore not be studied. Secondly, control subjects’ nodes present an equilibrium between being sources and sinks of information, or between *forcing* and *being forced*. Some of them, like C3 and C4 (motor cortex), and P3, Pz and P4 (parietal lobes, processing sensorial inputs) are mostly forced; this is to be expected, as these regions should not be active in an eyes closed resting state. On the other hand, the most *forcing* sensors are O1 and O2 in the occipital lobe, responsible for visual stimuli processing; and the frontal area. The existence of nodes being both sources and sinks of information could be explained by the presence of independent flows of information that have been linked to different frequencies in the brain activity^36,37^. Confirming such origin would nevertheless require a full band-dependent analysis; also comparing inter- and intra-groups variability, and using a larger number of clusters could yield a richer view of information transmission patterns.

Moving to the differences between both groups, these are represented in the right panel of Fig. 6. Green shades mark nodes that are less frequently sources in Schizophrenia patients, with the number inside them indicating the magnitude of the difference. Also, grey indicates nodes for which the difference between patients and control subjects was not statistically significant, according to a binomial test and for a significance level $\alpha = 0.01$. A global reduction in the *forcing* role is observed, which is in line with the disconnectivity observed in patients^38,39^. The only statistically significant exception to this tendency is P3, that is more frequently a source of information in patients; parietal nodes, including P3, have been related in the past to a deficient attribution of the source of control for intended actions^40,41^.

### Application: delay propagation patterns in air transport

The second real-world application here considered is a technological one, and specifically the analysis of delay propagation patterns in air transportation. Delay propagation is one of the most important research topics in air transport management, mainly due to the associated social, economical and environmental costs^42–44^. In order to analyse such propagations, the concept of functional networks has recently been proposed as a promising solution^12–14,45,46^, as it is based on the study of observable time series (in this case, time series of average delay at airports) without the need of a priori information about the underlying flight connectivity. I here consider the functional networks and data previously presented in Ref.^12^, focusing on the dynamics of the 25 largest European airports—see “Materials and methods” for details.

**Figure 7:**
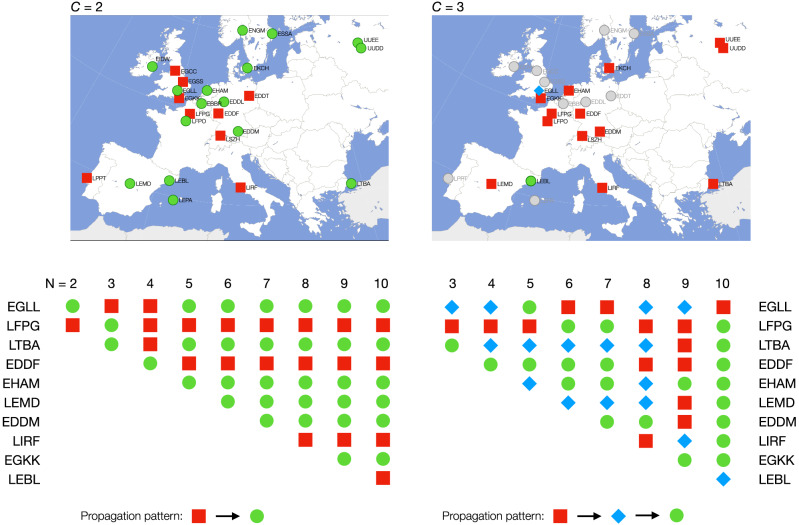
Analysis of delay propagation patterns in air transport. The top panels represent the clustering of the 25 largest European airports, respectively for $C=2$ (left) and $C=3$ (right). The color and shape of each airport represent its detected role, including net *forcing* (red squares), intermediary (blue diamonds, only for $C=3$) and net *forced* (green circles). Note that only the first 15 airports have been considered for $C=3$, due to the large computational cost; all other airports are marked in grey. Both maps were originally obtained from https://commons.wikimedia.org/wiki/File:Europe_polar_stereographic_Caucasus_Urals_boundary.svg with license https://creativecommons.org/licenses/by-sa/3.0/deed.en, and modified by using Keynote 11.0 software. The bottom panels report the evolution of the role of airports, as a function of the number of airports included in the analysis.

Figure 7 presents the assignment of each airport to the corresponding cluster, for $C=2$ (top left panel) and $C=3$ (top right panel). In the first case, airports are clustered in two groups: net *forcing*, i.e. here mostly propagating delays (red squares); and net *forced*, i.e. airports mostly receiving external delays (green circles). A structure seems to emerge, in which delay causing airports are located in the centre of Europe along a north-south axis—with the exception of Lisboa Portela Airport (LPPT). This may be due to how the central location of these airports also reflects in an *operational centrality*. Many airlines have their operational bases in these airports; any disruption there can then create delays that are propagated throughout the whole network. On the other hand, when an additional cluster is considered, the situation becomes more complex to be analysed. Specifically, the top right panel of Fig. 7 includes three types of nodes: mostly *forcing* (red squares), intermediaries (i.e. both receiving and propagating delays, blue diamonds), and mostly *forced* (green circles). In this case, results in Fig. 7 suggest that all but two airports are propagating their delays to London Heathrow airport (EGLL), and this latter to Barcelona (LEBL).

This example illustrates how the best solution for $C=3$ is not necessarily a (small) variation of the solution for $C=2$; due to the non-trivial way in which time series are aggregated, small changes in the initial conditions (number of elements, of clusters, etc.) can result in mayor changes in the result. This concept is further depicted in the bottom panels of Fig. 7, reporting the assignation of the top airports to the two or three clusters (for $C=2$ in the left side, and for $C=3$ in the right side) as a function of the number of considered airports. It can be appreciated that, firstly, adding an additional airport to a small set can completely change the resulting assignation; and, secondly, that an airport can have different (and even opposite) roles depending on the value of *C*.

**Figure 8:**
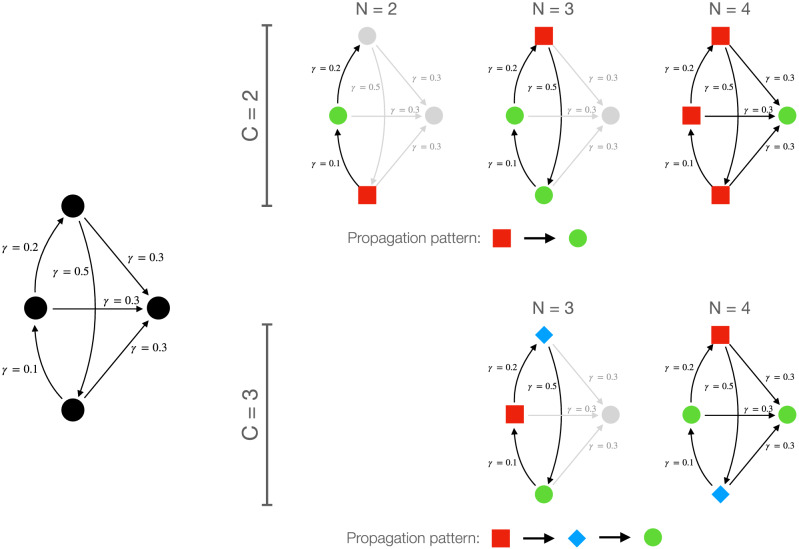
Evolution of the clustering with the size of the system. (Left) Graphical representation of a toy model composed of four dynamical units (nodes), pairwise linearly coupled (arrows). The number near each arrow indicates the corresponding coupling strength $\gamma$. (Right) Evolution of the best clustering when changing the number of analysed nodes *N* (from left to right), and the number of clusters *C* (from top to bottom). Node colour and shape represent the detected role, including net *forcing* (red squares), intermediary (blue diamonds, only for $C=3$) and net *forced* (green circles).

In order to exemplify how such apparent instability of the solution emerges, Fig. 8 (left side) presents a simple toy model composed of four dynamical systems linearly coupled between them—i.e. equivalent to the model of Eq. (5). The right part of the figure further depicts the best solutions obtained by increasing the number of nodes (from left to right), and by increasing the number of clusters (from top, $C=2$, to bottom, $C=3$). In the simplest case of $N=2$ and $C=2$, the solution is trivial and only implies detecting the direction of the causality. When a third node is added, the strongest link becomes the one connecting the top to the bottom node, and the clustering reflects this by merging the middle and bottom nodes in the *forced* cluster. Finally, when all nodes are considered, the structure once again changes to reflect the main left-to-right flow of information. This illustrates how nodes can drastically change their role when new elements are included in the analysis; this is nevertheless not an instability of the proposed approach, but rather a reflection of how macroscopic information flows are the non-trivial result of microscopic ones.

### Computational cost and approximate solutions

**Figure 9:**
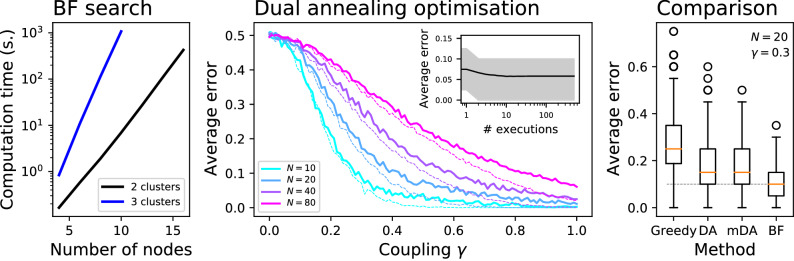
Computation cost and optimisations. (Left) Evolution of the computation cost of the brute force algorithm as a function of the number of nodes to be clustered, for $C=2$ (black line) and $C=3$ (blue line). The cost is measured in seconds, calculated on an Apple iMac with an hex-core Intel Core i5 at 3.3 GHz and executed on a single core. (Center) Average error obtained by a dual annealing optimisation, for $C=2$, time series composed of 500 values, and as a function of the coupling constant $\gamma$ and the number of nodes *N*. Solid lines represent results for one single execution of the optimisation algorithm, while dashed lines the best solution, measured as the one with the smallest *J*, among 50 executions with random initial conditions. The inset represents the evolution of the error as a function of the number of executions of the dual annealing optimisation, for $N=40$ and $\gamma = 0.6$; the grey band depicts the evolution of the 10-90 percentile interval over $10^3$ realisations. (Right) Distribution of the errors, for $N=20$, $C=2$ and $\gamma = 0.3$, obtained by four algorithms: from left to right, greedy optimisation, dual annealing (DA) optimisation, dual annealing optimisations with 50 executions (mDA), and brute force (BF). Horizontal orange lines depict the median of each distribution and circles their outliers.

As previously shown, the complexity of a brute force algorithm exploring the complete parameter space is $O(C^N)$. This implies that this approach is feasible only on small networks, as the time required to analyse a system composed of 12 elements already exceeds one minute for two clusters, and one hour for three clusters—see Fig. 9, left panel, times calculated with a 3.3 GHz Intel Core i5 using a single core. Larger networks, e.g. up to 30 nodes, can still be analysed taking advantage of a parallelisation approach, i.e. by dividing the search space into non-overlapping regions. To illustrate, the problem can be split in two by executing the optimisation twice, by assigning the first element to respectively cluster one and two, and by then choosing the best solution.

In a way similar to clustering analysis in data mining^47,48^, finding solutions for large-scale data sets require the use of some heuristic, i.e. of algorithms assuming some structure in the data and yielding approximate (but still useful) results. These may include, for instance, a greedy optimisation strategy, which firstly optimises the cluster assignment of half of the elements; for then completing the task, by considering the solution found for the first half as fixed. An alternative solution may be represented by stochastic optimisation algorithms, which are based on stochastically improving an initial solution by selecting elements at random, switching their assigned cluster, and retaining the new solution if a lower *J* is achieved. While exhaustively exploring all these alternatives is outside the scope of this work, and will require joining expertises from different fields of the scientific community, I here evaluate the use of a standard dual annealing optimisation algorithm^49^. It is the result of combining a classical simulated annealing optimisation^50^ with a local search on accepted locations, thus yielding more refined solutions than what usually obtained by a simple annealing. For the sake of simplicity, the standard Python implementation included in the SciPy library^51^ has been used.

The average error incurred by the dual annealing algorithm can be seen in Fig. 9 (central panel, solid lines), for sets of *N* time series linearly coupled as in Fig. 2, and as a function of the coupling constant $\gamma$. As may be expected, the error is higher for large systems, i.e. for large values of *N*; still, these approximated solutions are obtained in seconds, even for *N* as large as 80—a scenario impossible to tackle with a brute force search. Errors can also be reduced by performing the optimisation multiple times, starting from random initial conditions, for then selecting the result with the minimum *J*. This results in a minor reduction of the error (see dashed lines of Fig. 9 central panel, for 50 random repetitions, and also the inset in the same panel), in exchange for a linear increase in the computation cost.

The right panel of Fig. 9 finally reports a box plot of the distribution of the errors obtained by four algorithms, namely the previously-described greedy one, the dual annealing optimisation (DA), the annealing optimisation executed 50 times (mDA), and the brute force (BF) one (for $N=20$ and $\gamma = 0.3$). It can be appreciated that both dual annealing optimisations yield results close to the optimal solution found by the brute force approach, in terms of the medians of the distributions; they nevertheless also present a large dispersion and a larger number of outliers.

Finally, it is worth noting that the errors reported in Fig. 9 are the result of two contributions: the error derived from a wrong estimation of the Granger test *p*-value, due to the finiteness of the time series; and the additional error introduced by the use of an optimisation algorithm. To illustrate, the error obtained for $N = 80$ and $\gamma = 0.2$ by the dual annealing optimisation is $0.361 \pm 0.076$ (mean and standard deviation over 100 random realisations) for time series of length $10^3$, but it drops to $0.278 \pm 0.058$ for $2 \cdot 10^3$, $0.168 \pm 0.054$ for $4 \cdot 10^3$ and to $0.117 \pm 0.048$ for $8 \cdot 10^3$. Excellent estimations can thus be obtained, provided long time series can be secured.

## Discussion and conclusions

Functional networks have become a powerful instrument for the analysis of complex systems, as they allow recovering the underlying connectivity structure through the analysis of the elements’ dynamics. When reconstructed through causality metrics, these networks provide a detailed picture of the information flows within the system; yet, at the same time, extracting a macroscopic synthesis of these flows is not always simple. In other words, functional networks are good representations of the trees, but not of the overall forest.

In this contribution I propose an adaptation of machine learning’s clustering analysis^47,48^ to functional networks. Nodes are grouped according to their role in the global information flow, which is matched against a desired connectivity motif. The result is a simplified representation of the global structure, able for instance to highlight which nodes are sources and which ones are sinks of information—or, from a Granger causality perspective, which nodes are mostly *forcing* or *being forced*.

The causality clustering here presented can be expanded in several directions. On one hand, the attentive reader would have noticed that, while the idea of causal clusters has here been illustrated through the celebrated Granger causality, almost any other directed causality metric can be used. The simplest case include those metrics whose output is a *p*-value, which could directly be introduced in Eq. (4)—as for instance frequency-based Granger tests^52^. On the other hand, causality metrics yielding a *strength* (e.g. transfer entropy^53^) can also be used, provided Eq. (4) is adapted accordingly—i.e. the strength has to maximised, as opposed to the *p*-value that has to be minimised. On the other hand, causality patterns of any size, i.e. not limited to $C=2$ and 3, can be evaluated. For that one only needs to define a suitable matrix *M* and optimise the cluster assignation in order to minimise *J* in Eq. (4). Still, one should also be aware of the increased computational cost.

The large computational cost is actually one of the main problems of the proposed approach. A brute force optimisation of the cost function *J* has a complexity scaling as $C^N$, with *C* and *N* respectively being the number of clusters and elements (nodes). This implies that a brute force approach is feasible only for systems composed of 20–30 elements. In case of larger data sets, one must resort to heuristics yielding approximate results. As shown in Fig. 9, a dual annealing optimisation^49^ can achieve acceptable error rates at a fraction of the original computational cost. Clearly, the applicability of this method to other real-world problems will depend on the development of more optimised and efficient algorithms.

One may also list a certain complexity of this approach among its drawbacks. Specifically, as shown in Figs. 7 and 8, results can strongly vary when *C* or *N* are changed, such that adding one additional element can change the role assigned to the other elements of the system. This is nevertheless not due to an instability of the algorithm, whose solutions are stable for simple systems as the one of Eq. (5); on the contrary, this is the reflection of the complexity of the underlying dynamics, as illustrated in the toy example of Fig. 8. A future line of research will involve applying the proposed approach to describe the multi-scale evolution of causality, and how local interactions are modified by global information.

## Materials and methods

### Python library

A Python library implementing the causality clustering here described is freely available at https://gitlab.com/MZanin/causality-clustering. It includes a function to calculate *J* given a set of time series, plus functions to perform brute-force and dual annealing searches. Additional files include examples using synthetic data, and a unit testing suit.

### EEG recordings

The electroencephalographic (EEG) recordings here used correspond to a set of schizophrenia patients and matched control subjects, as described in Ref.^54^ and available at http://dx.doi.org/10.18150/repod.0107441. The 14 patients (7 males, $27.9 \pm 3.3$ years, and 7 females, $28.3 \pm 4.1$ years) met International Classification of Diseases ICD-10 criteria for paranoid schizophrenia (category F20.0). The 14 corresponding healthy controls were 7 males, age of $26.8 \pm 2.9$ years, and 7 females, age of $28.7 \pm 3.4$. Fifteen minutes of EEG data were recorded during an eyes-closed resting state condition. Data were acquired at 250 Hz using the standard 10–20 EEG montage with 19 EEG channels: Fp1, Fp2, F7, F3, Fz, F4, F8, T3, C3, Cz, C4, T4, T5, P3, Pz, P4, T6, O1, O2. The reference electrode was placed at FCz. All recordings have been split in sub-windows of 2000 points, i.e. representing 8 s each. For each subject, 15 sub-windows have been used in the analysis, taken as independent trials, yielding a total of 210 sets of time series for each group. The Granger causality has been calculated between each pair of time series using the broadband signal, using a maximum lag of 15 points (corresponding to 60 ms). No additional preprocessing (e.g. artefact removal) has been performed.

### Air traffic data

This data set includes time series of average delays at the 50 largest European airports, as described in Ref.^12^. These time series have been obtained by analysing aircraft trajectories included in the Flight Trajectory (ALL-FT+) data set provided by the EUROCONTROL’s PRISME group. It includes information about planned and executed trajectories for all flights crossing the European airspace, with positions reported on average every 2 min. The data set covers the period from 1st March to the 31st December 2011, including a total of $10.3 \cdot 10^6$ flights. Only flights landing at the 25 busiest European airports (in terms of number of operations) have further been processed.

A time series has been extracted for each airport, representing the average hourly delay of arriving flights. Delays are here calculated as the difference between actual and planned landing time, and as such can also be negative (when an aircraft arrived before time). Due to missing data, each time series comprises 7440 values. These time series are characterised by a significant non-stationarity, as delays are strongly correlated to traffic volumes—i.e. they are higher during peak hours, week days and the summer. In order to reduce biases in the calculation of the causality, a detrend process has then been performed, by subtracting the average delay observed in the same day, in the two previous and following weeks, at the same hour, i.e.:

6 $$\begin{aligned} {{\bar{d}}}(t) = d(t) - \frac{1}{4} \sum \limits _{i \in \{ - 2, - 1,1, - 2\} } {d(t + 168i)}, \end{aligned}$$

*d*(*t*) being the original time series at time *t*, and ${{\bar{d}}}(t)$ the final time series. According to this definition, ${{\bar{d}}}(t)$ thus represents the difference between the observed and the expected (historical) delay.
